# Major Histocompatibility Complex (MHC) Class I and MHC Class II Proteins: Conformational Plasticity in Antigen Presentation

**DOI:** 10.3389/fimmu.2017.00292

**Published:** 2017-03-17

**Authors:** Marek Wieczorek, Esam T. Abualrous, Jana Sticht, Miguel Álvaro-Benito, Sebastian Stolzenberg, Frank Noé, Christian Freund

**Affiliations:** ^1^Protein Biochemistry, Institute for Biochemistry, Freie Universität Berlin, Berlin, Germany; ^2^Computational Molecular Biology Group, Institute for Mathematics, Berlin, Germany

**Keywords:** antigen presentation, major histocompatibility complex, HLA, protein dynamics, peptide exchange, tapasin, HLA-DM, adaptive immunity

## Abstract

Antigen presentation by major histocompatibility complex (MHC) proteins is essential for adaptive immunity. Prior to presentation, peptides need to be generated from proteins that are either produced by the cell’s own translational machinery or that are funneled into the endo-lysosomal vesicular system. The prolonged interaction between a T cell receptor and specific pMHC complexes, after an extensive search process in secondary lymphatic organs, eventually triggers T cells to proliferate and to mount a specific cellular immune response. Once processed, the peptide repertoire presented by MHC proteins largely depends on structural features of the binding groove of each particular MHC allelic variant. Additionally, two peptide editors—tapasin for class I and HLA-DM for class II—contribute to the shaping of the presented peptidome by favoring the binding of high-affinity antigens. Although there is a vast amount of biochemical and structural information, the mechanism of the catalyzed peptide exchange for MHC class I and class II proteins still remains controversial, and it is not well understood why certain MHC allelic variants are more susceptible to peptide editing than others. Recent studies predict a high impact of protein intermediate states on MHC allele-specific peptide presentation, which implies a profound influence of MHC dynamics on the phenomenon of immunodominance and the development of autoimmune diseases. Here, we review the recent literature that describe MHC class I and II dynamics from a theoretical and experimental point of view and we highlight the similarities between MHC class I and class II dynamics despite the distinct functions they fulfill in adaptive immunity.

## Introduction

Major histocompatibility complex (MHC) class I and class II proteins play a pivotal role in the adaptive branch of the immune system. Both classes of proteins share the task of presenting peptides on the cell surface for recognition by T cells. Immunogenic peptide–MHC class I (pMHCI) complexes are presented on nucleated cells and are recognized by cytotoxic CD8+ T cells. The presentation of pMHCII by antigen-presenting cells [e.g., dendritic cells (DCs), macrophages, or B cells], on the other hand, can activate CD4+ T cells, leading to the coordination and regulation of effector cells. In all cases, it is a clonotypic T cell receptor that interacts with a given pMHC complex, potentially leading to sustained cell:cell contact formation and T cell activation.

**Figure 1:**
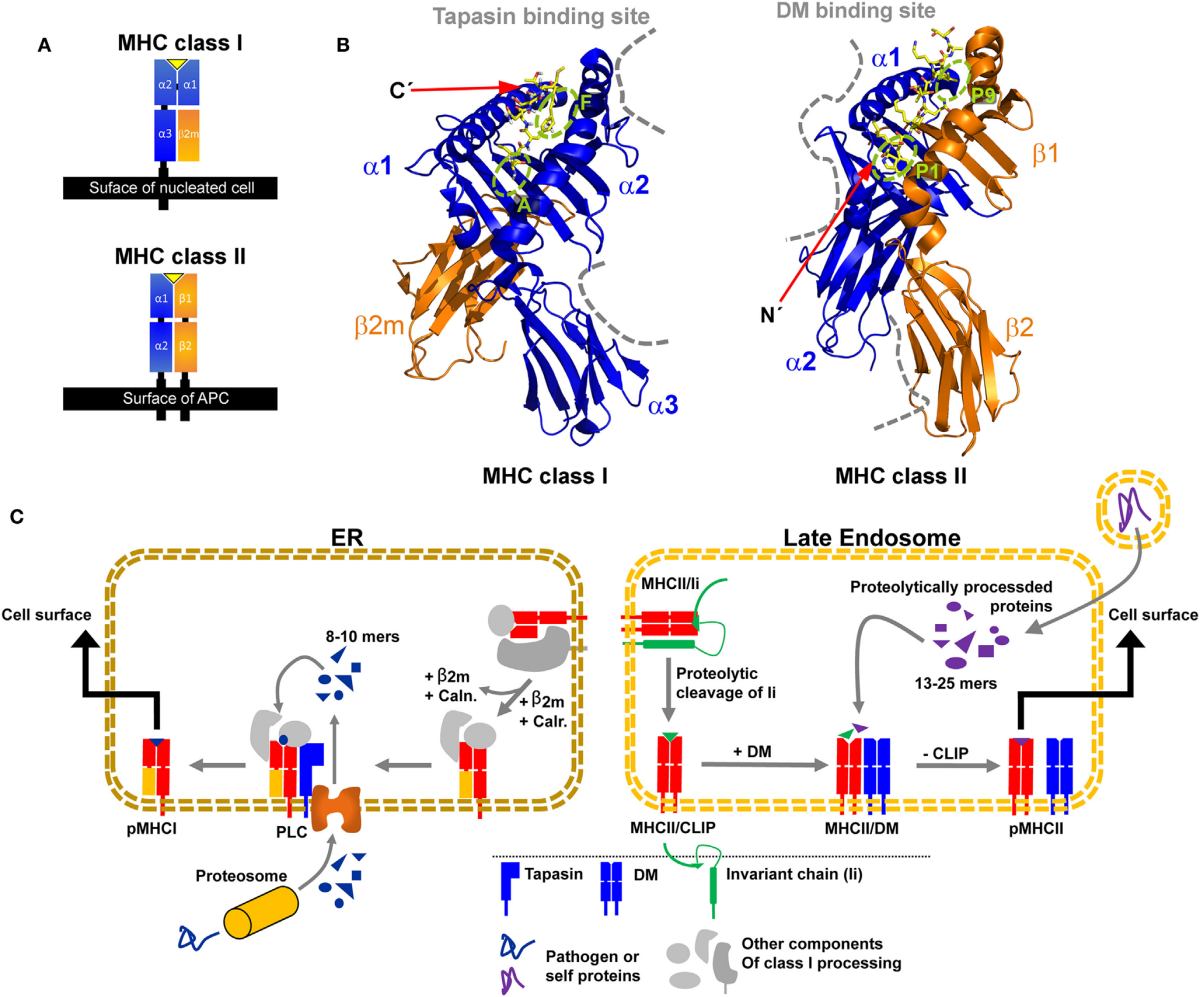
**Structural characteristics of major histocompatibility complex (MHC) class I and MHC class II proteins and their compartment-dependent loading with processed peptides**. **(A)** Domain topology of a pMHC class I and pMHC class II complex. **(B)** Structure of HLA-A68 in complex with an HIV-derived peptide (PDB: 4HWZ, left) and HLA-DR1 in complex with a hemagglutinin-derived peptide (1DLH, right). Indicated are the supposed interaction sites of MHC class I with tapasin and of MHC class II with DM as dashed gray lines. The peptide is shown in yellow with its N and C-terminus marked and relevant pockets are labeled green **(C)** Simplified illustration of MHC class I (left) and II (right) processing and peptide-editing pathways. CLIP, class II-associated invariant chain peptide; Caln., calnexin; Calr., calreticulin; ER, endoplasmic reticulum; PLC, peptide loading complex.

Major histocompatibility complex class I and class II share an overall similar fold. The binding platform is composed of two domains, originating from a single heavy α-chain (HC) in the case of MHC class I and from two chains in the case of MHC class II (α-chain and β-chain) (Figure 1A). The two domains evolved to form a slightly curved β-sheet as a base and two α-helices on top, which are far enough apart to accommodate a peptide chain in-between. Two membrane-proximal immunoglobulin (Ig) domains support the peptide-binding unit. One Ig domain is present in each chain of MHC class II, while the second Ig-type domain of MHC class I is provided by non-covalent association of the invariant light chain beta-2 microglobulin (β_2_m) with the HC. Transmembrane helices anchor the HC of MHC class I and both chains of MHC class II in the membrane (Figure 1A).

The groove in-between the two helices accommodates peptides based on (i) the formation of a set of conserved hydrogen bonds between the side-chains of the MHC molecule and the backbone of the peptide and (ii) the occupation of defined pockets by peptide side chains (anchor residues P2 or P5/6 and PΩ in MHC class I and P1, P4, P6, and P9 in MHC class II) (1–4). The type of interactions of individual peptide side-chains with the MHC depend on the geometry, charge distribution, and hydrophobicity of the binding groove. Predicting the affinity of these distinct MHC–antigen interactions for individual allotypes has been a long-standing goal in the community. While good progress has been made in developing and optimizing bioinformatic algorithms to estimate peptide binding to MHC proteins, these *in silico* predictions, however, still yield false positives (5, 6), and often fail in predicting immunodominance. We argue that understanding the relevance of transient or energetically excited protein conformations that are visited during the equilibrium fluctuations of the molecular structure is important for making good predictions.

In MHC class I, the binding groove is closed at both ends by conserved tyrosine residues leading to a size restriction of the bound peptides to usually 8–10 residues with its C-terminal end docking into the F-pocket (7–9). In contrast, MHC class II proteins usually accommodate peptides of 13–25 residues in length in their open binding groove, with the peptide N-terminus usually extruding from the P1 pocket (10). It has been reported that the interactions at the F pocket region in MHC class I and the P1 region (including the P2 site) in MHC class II appear to have a dominant effect on the presentation of stable pMHC complexes and on the immunodominance of certain peptidic epitopes (11–16). Interestingly, these pockets are located at opposite ends of the binding groove of the respective MHC class I and MHC class II structures (Figure 1B).

The most polymorphic human MHC class I and class II proteins (human leukocyte antigens, HLAs) are each expressed from three gene regions (MHC class I: HLA-A, -B, -C; MHC class II: HLA-DR, -DP, -DQ), which are all highly polymorphic. This allelic variation mainly affects the nature and composition of the peptide-binding groove and thus modulates the peptide repertoire that is presented on the surface by MHC class I or MHC class II proteins for CD8+ or CD4+ T cell recognition, respectively. A good match of the peptide and the MHC binding groove is an important, but certainly not the sole determinant of its presentation. In fact, the formation of a pMHC complex depends on its peptide-loading pathway, in which the selection of peptides is influenced by several factors, such as antigen availability, protease activity, or the availability of chaperones. In addition, for each MHC class, a “catalyst” is available to enhance peptide exchange for certain peptides: tapasin for MHC class I and HLA-DM for MHC class II. These molecules edit the presented peptide repertoire and bias the exchange reaction toward the presentation of thermodynamically stable complexes. Tapasin and HLA-DM thus act similar to typical enzymes by reducing the energy barrier for peptide exchange. However, in the case of HLA-DM and tapasin, no covalent bonds are formed or cleaved during the exchange reaction.

The MHC class I HC folds and assembles with β_2_m in the lumen of the endoplasmic reticulum (ER). The partially folded heterodimer is then incorporated into the peptide-loading complex (PLC) for peptide binding and exchange. In the PLC, tapasin is a protein that catalyzes, together with other chaperones, the loading of high-affinity peptides derived from proteolysis of endogenously expressed proteins (Figure 1C, left panel) (17, 18). In the absence of tapasin, some class I allotypes (such as HLA-B*44:02) are retained in the ER (tapasin-dependent), whereas other class I proteins (tapasin-independent, such as HLA-B*44:05 and HLA-B*27:09) can bind peptides and travel to the cell surface (19–22). There is no crystal structure of the MHC class I/tapasin complex, but several structural models and mutational studies suggested that tapasin binds two regions in the HC of MHC class I, a loop in the α_3_ domain (residues 222–229), and a region of the α_2_ domain (residues 128–137) adjacent to the F-pocket (Figure 1B) (18, 21, 23–29).

Major histocompatibility complex class II proteins fold in the ER in complex with a protein called invariant chain (Ii) (30) and are then transported to late endosomal compartments (also coined MHC class II compartment, MIIC). There, Ii is cleaved by cathepsin proteases and a short fragment remains bound to the peptide-binding groove of MHC class II proteins, termed class II-associated invariant chain peptide (CLIP). This placeholder peptide is then normally exchanged against higher affinity peptides, which are derived from proteolytically degraded proteins available in endocytic compartments (Figure 1C, right panel). HLA-DM accelerates peptide exchange, with different allelic variants being more or less susceptible to catalysis. HLA-DM has a highly similar structural fold compared to classical MHC class II proteins, but its closed-up binding groove prevents peptide binding. Crystal structures of HLA-DM in complex with the MHC class II protein HLA-DR1 (31) and in complex with the competitive inhibitor HLA-DO (32) revealed that HLA-DM mainly contacts the α_1_-domain of MHC class II proteins close to the P1 pocket and additionally the membrane-proximal β2-domain, in line with previous mutational analyses (Figure 1B) (13, 33–35).

Despite the structural differences between tapasin and HLA-DM as well as their presumably opposite sites of interaction with regard to the orientation of the binding groove, a similar mode of action has been suggested, hinting at a possible convergent evolution of the two exchange catalysts (Figure 1B) (36). A common feature seems to be that both catalysts target regions in the vicinity of those pockets in the peptide-binding groove that are of great relevance for the stability of the respective pMHC complex (11, 13, 15, 31, 37). Furthermore, in both cases, the binding of a high-affinity peptide is able to release the interaction with tapasin/DM (13, 26, 38, 39) and allows for transport of stable pMHC complexes to the cell surface.

While the general hallmarks of antigen processing and editing have been established, the discussion is now moving toward the dynamics of the system, both at the cellular and molecular level. The mechanistic questions relate to a description of how exactly peptides are selected for presentation and how tapasin and HLA-DM catalyze this reaction in an allele-specific manner.

## Structural Variations in MHC Complexes

Many allelic variants of MHC class I and MHC class II bound to individual peptide antigens display different biochemical features, but surprisingly, their “ground-state,” i.e., thermodynamically most stable conformations reported by the many available pMHC X-ray structures are very similar. In contrast, increasing experimental and computational evidence of wild type (WT) and mutant MHC complexes over the past years incontestably revealed that changes in conformational dynamics in MHC proteins have to accompany peptide loading and exchange (22, 40–46).

**Figure 2:**
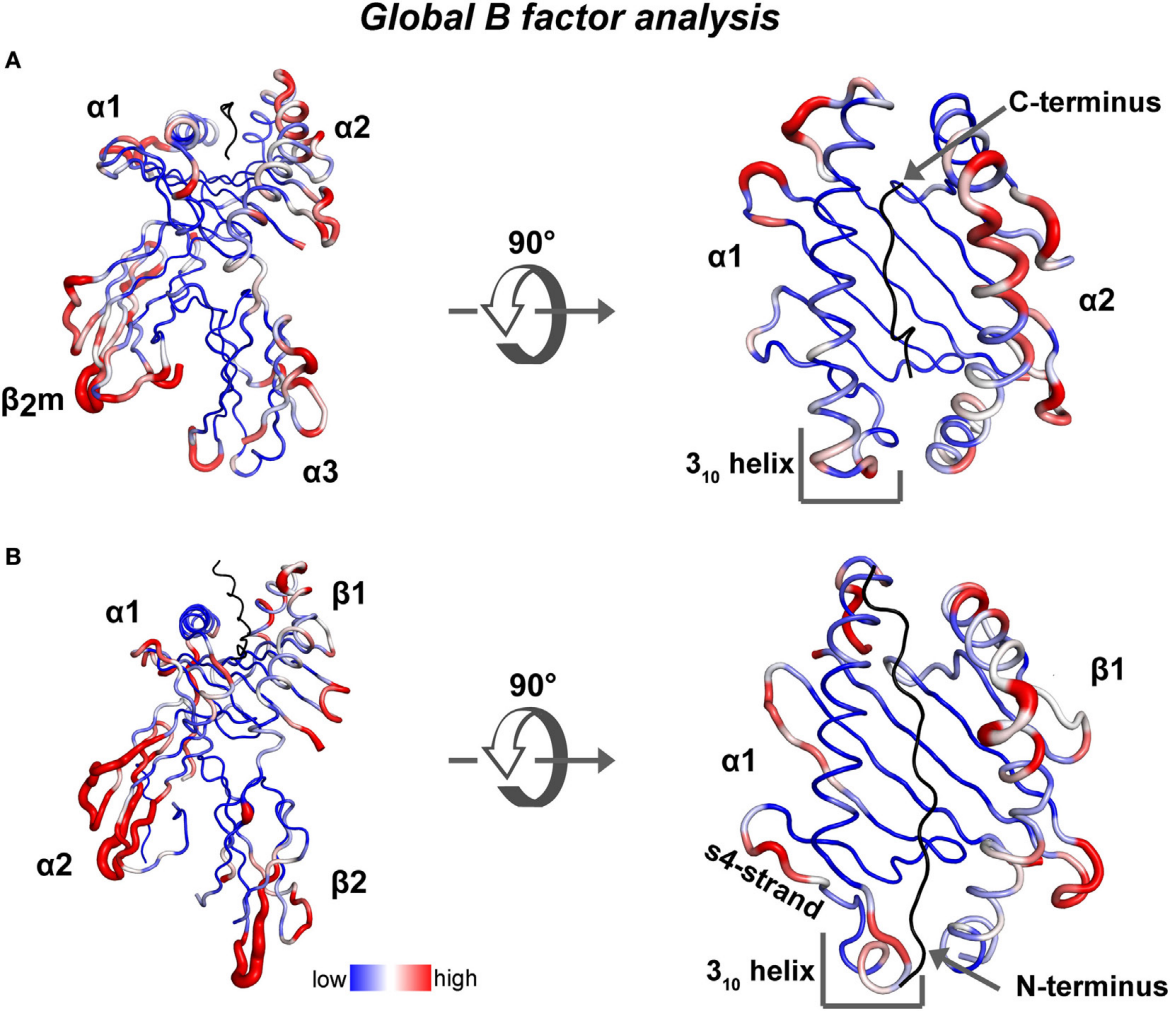
**Global B-factor analysis of X-ray crystal structures of MHC class I and MHC class II**. Shown is the variance of the normalized residual B factor values of CA atoms **(A)** derived from 297 human pMHC class I structures is plotted as blue to red spectrum on a HLA-A*0201/peptide complex (PDB: 5HHN) and **(B)** from 41 human pMHC class II structures is plotted on a DR1/peptide complex (PDB: 4X5W).

To highlight possible dynamic regions within ground-state crystal structures of human MHC class I and class II proteins bound to a peptide, we performed a global B-factor analysis of all available X-ray crystal structures of human MHC complexes in the absence of any other binding partner. In each structure, we normalized the B-factor values of each alpha carbon (CA) atom to the global mean. Then the variance of all the normalized B-factor values for each CA atom, in 297 human pMHC class I and 41 human pMHC class II structures, was calculated and depicted with a blue to red color spectrum, respectively (heat map on structures of HLA-A*0201 and HLA-DR1, Figures 2A,B). Overall in the binding groove of class I (class II), the α-helix in the α2 (β1) domain displays higher B-factor variation values than the α-helix in the α1 (α1) domain and the β-strands from both domains. Among the pMHCI structures, although B-factor variation values in the N-terminus-proximal helical segments indicate the existence of a certain degree of dynamics, the α2-helical region around the peptide C-terminus displays the highest variation in B-factors (Figure 2A). Among the class II structures, high B-factor variation values are found especially in β-strands 2 and 4 of the α-chain, the 3_10_ helical region and almost the entire β-chain α-helix (Figure 2B). Our analysis is corroborated by a previous comparison of 91 different pMHC class II crystal structures (47). In this analysis, conformational heterogeneities were observed in three regions: the 3_10_-helical region (α45–54), the kink region in the β1-helix (β62–71), and the β2-domain (β105–112).

It is known that, to some extent, structural variations can be introduced by variable peptide-binding modes. In this context, peptides longer than 8–10 residues have been reported to bind to the MHC class I binding groove (48–52). To accommodate the increase in length, the peptides have to bulge out, leaving the central residues (between p2 and pΩ) exposed to solvent. This is usually achieved by a kink in the backbone in the middle part of the peptide. Recently, two crystal structures of HLA-A2 bound to15-mer peptides have been solved (53). The two peptides follow a binding mode similar to that of the canonical peptides with two anchor residues in the B and F pockets. While one of the peptides showed a mobile central conformation similar to another reported long peptide (48, 54), the other peptide adopted an unusual rigid β-hairpin secondary structure. Furthermore, although the binding of the N- and C-termini at both ends of the binding groove is conserved in almost all the MHC class I complexes, some exceptions have been reported. For example, in the F pocket of HLA-A2, the C terminal residue of the peptide extends by ~1 Å leading to a significant rearrangement of the pocket with only one of the standard hydrogen bonds (at Thr143) preserved (55). Another example is seen in the HLA-B35 protein, the short N-terminus of the 8-mer peptide does not reach the A pocket. Instead, the hydrogen bonds between the amino group of P1 residue and residue 45 of MHC class I are mediated by a water molecule (56).

Since, in the case of MHC class II proteins, the peptide ligand within the binding groove usually adopts a pseudosymmetrical PPII helix-like conformation, bidirectional binding is theoretically possible. An interesting case represents a crystal structure of a DR1/CLIP complex, in which the peptide binds in a very unusual, inverted orientation (C-terminus close to the P1 pocket). The driving force for this peptide inversion is the formation of three additional H-bonds of P1-close residues and the backbone of the peptide’s C-terminus (57, 58). Biological and biochemical evidence for the existence of other pMHC class II isomers have been described in the context of autoimmunity (59). Since MHC class II proteins have open binding grooves, peptides can protrude outwards and even bind in different registers. In this regard, an insulin B chain-derived peptide (InsB_9–23_) was suggested to induce type 1 diabetes (T1D) in a thermodynamically less favored, low-affinity binding register (60, 61).

**Figure 3:**
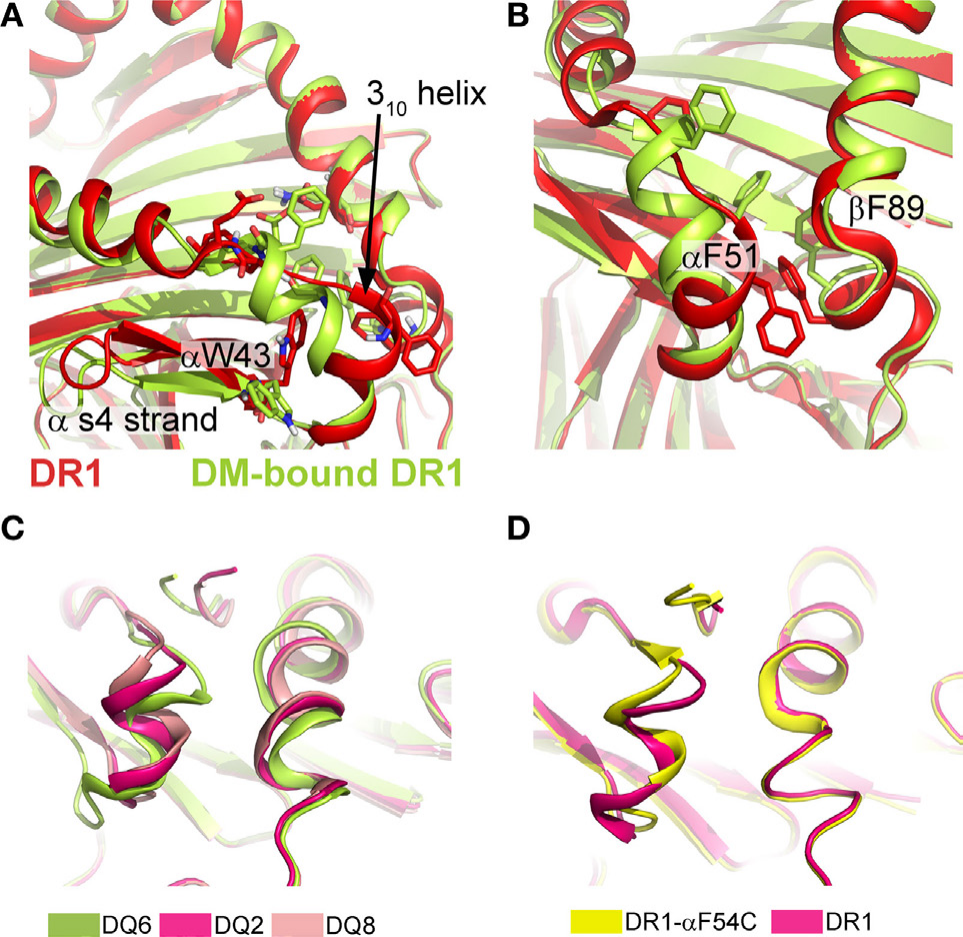
**Conformational rearrangements upon DM binding and structural variations in type 1 diabetes-susceptible DQ complexes**. **(A)** Structural rearrangement in the α1-S4 strand and 3_10_-helical region seen in DR1 when bound to DM (limon cartoon) compared to DR1 unbound DM (red). **(B)** DM-induced rearrangements in the P1-pocket and the surrounding helical segments. PDBs used in **(A,B)** 1DLH and 4FQX. **(C)** Overlay of DQ2/ag (PDB: 1S9V), DQ6/hyp_1–13_ (PDB:1UVQ) and DQ8/InsB_9–23_ (PDB: 1JK8) showing the structural variations of the 3_10_ helix and the P1-proximal β1-helix. Interdomain communication as exemplarily indicated by the hydrogen bond between αR52 and βE86/βT89 in the DQ8 allele variant is thought to increase the stability of these regions and was previously discussed to be linked to a lowered DM-susceptibility (62, 63). ag, αI-gliadin; hyp, hypocretin peptide 1–13; InsB, insulin B chain 9–23. **(D)** Structural alignment of DR1/CLIP (PDB: 3QXA) and DR1-αF54C/CLIP (PDB: 3QXD), a mutant that shows an altered conformation in the 3_10_ helix and an increased DM susceptibility.

Apart from variable peptide binding, catalyst binding can induce more significant conformational variation, as seen in DM-bound DR. By designing a P1-anchor-free pMHC class II complex, Pos et al. could increase the affinity for the pMHC class II to DM and solve the crystal structure for the covalently tethered DR1/HA/DM complex (31). In this MHC class II/DM complex, the interaction interface is primarily composed by the α-subunits of DM and DR1 (~65% of the entire interaction surface, Figure 1B). DM binding to DR stabilizes a rearranged conformation in the vicinity of the P1 pocket of DR1 resulting from the absence of critical peptide-MHC class II interactions in this region. The extended region in the DR1α-chain (α52–55) and the 3_10_-helix adopt an α-helical fold (Figure 3A). Compared to other parts of the pMHC class II structure, it was shown that this site indeed represents a conformationally labile region (35, 46). In addition, the C-terminal part of the β1-α-helix (β86–91) becomes slightly less structured (Figure 3B).

A feature, which is also present in DM-bound DO, is the intermolecular H-bonds between two conserved residues (DRα W43 and DMα N125). The formation of these critical bonds (as well as other interactions) likely depends on the “flipping out” movement of αW43 from the P1 pocket of DR1 toward DMα (Figure 3A). This movement of DR1 αW43 was suggested to be triggered by partial dissociation of the peptide’s N-terminus or by transient destabilization of contacts of the peptide N-terminus when bound to MHC class II (13, 64). As a consequence, the P1 site is stabilized by a repositioning of two phenylalanine sidechains (DRα F51 and DRβ F89), thereby compensating for the loss of peptide anchors and αW43 from this region (Figure 3B). Incoming peptides have to compete with these repositioned Phe residues for the P1/P2 site in order to be selected for display. Interestingly, structural characteristics of T1D-conferring HLA-DQ alleles were indeed discussed to be linked to decreased DM-sensitivity (62). The analysis of several DQ variants indicated structural differences of the T1D-risk variants DQ2 and DQ8 when compared to DQ1, DQ 6, or DR variants. The decrease in DM-susceptibility of these two proteins was explained by a stabilization of the 3_10_ helical region (63) (Figure 3C). However, the exact relationship between structural variations in the 3_10_ helix and DM-susceptibility are not clear, as highly DM-susceptible DR complexes can display a different conformational mode, compared to DM-susceptible DQ alleles (Figure 3D).

The observed changes lead to the question as to how much structural plasticity in these regions preexists in the peptide-loaded form and provide a prerequisite for catalyzed as well as for spontaneous peptide exchange. For example, do pMHC complexes sample conformations observed in simulations of empty proteins or in complex with the catalyst? How would allelic variation affect the distribution of MHC proteins within the conformational space and thereby influence the presented peptide repertoire? Could variation in protein plasticity also account for the association of specific MHC alleles with immune diseases? Since the polymorphic peptide-binding groove of MHC proteins defines its affinity for a certain peptide, substitutions of even a single amino acid may lead to significantly different affinities for individual peptides. In general, the critical factors defining whether a peptide is presented or not are determined at the different levels of antigen processing and presentation such as uptake route, amount, and folding state of the antigenic protein, amenability to proteolytic degradation, and catalysis of the complex, etc. However, at the molecular level, it has been shown that certain polymorphisms shape individual pockets in the peptide-binding groove to optimally present an autoimmunogenic self-peptide (65–67). In other cases, the functional impact of disease-associated polymorphisms remained enigmatic and suggests that dynamics might account for the observed differences.

## Dynamics of Peptide-Free MHC Proteins

While simulations and experimental studies vary in the features ascribed to peptide-free MHC proteins, they certainly agree in attributing a substantial degree of dynamics to the peptide-binding groove. Thus, binding of peptides to MHC proteins is of utmost importance for the stabilization of the known MHC fold (40, 68). The lack of a crystal or an NMR structure of peptide-free MHC protein hinders an accurate description of the structural changes upon peptide binding and this is probably due to the ensemble character of the peptide-free conformers. This ensemble character, however, has been probed by computational techniques, as discussed in the following paragraphs.

### MHC Class I

Most of the conformational dynamics information on the peptide-free class I have been revealed by molecular dynamics (MD) simulations. In such simulations, peptide-free class I protein is modeled from the crystal structure by deleting the atoms of the bound peptide. In the absence of peptide, an increased conformational flexibility of the F pocket region was observed for several allelic variants (HLA-A*02:01, HLA-B*44:02, HLA-B*44:05, HLA-B*27:05, HLA-B*27:09, H-2D^b^, and H-2K^b^) (9, 15, 21, 22, 69–71). Longer simulations of chicken and human class I allotypes showed increased global motion in the peptide-free form when compared to the peptide-bound proteins (72, 73). By combining molecular docking and MD simulations, a conformational transition of the 3_10_ helical segment of H-2L^d^ between the peptide-bound and peptide-free class I was observed. Thus, a conformational reorganization close to the A and B pockets upon peptide binding was proposed (74).

Experimentally, circular dichorism (CD) was used to measure the thermal denaturation temperature (T_m_) of the peptide-free HLA-B*07:02 (B7/β2m). By increasing the temperature, a gradual loss of the structure-specific signal of B7/β_2_m in the CD spectrum of peptide-free class I was detected, indicating a more heterogeneous conformational population. Furthermore, in the absence of peptide, the binding groove of B7/β_2_m was more sensitive to enzymatic proteolysis when compared to the peptide-bound form (40). Saini et al. studied the unfolding of H-2K^b^ by measuring the intrinsic tryptophan florescence. The results pointed to a folding intermediate of peptide-free class I proteins that are more structured than a molten globule (75). This was in line with a previous study arguing for a native-like conformation of *in vitro* refolded empty murine class I proteins (76).

Structural studies using NMR indicated a loss of the binding-groove fold in the peptide-free form of a HLA-C allotype. In particular, NMR spectra of these peptide-free MHC class I protein show the loss of selected methionine NH resonances of the β-sheet floor of the peptide-binding groove, which indicated unfolding or conformational exchange of this part of the protein (77). This is consistent with previous reports indicating that especially the binding groove is undergoing conformational exchange in the absence of the bound peptide (40, 78).

A work that focused on complexes with a partly filled MHC class I binding groove pointed to a requirement of the stabilization of the F pocket region. By using a refolded H-2D^b^ in the presence of a pentamer peptide (NYPAL), which binds to the C to F pockets in the binding groove, a X-ray crystal structure of the pMHCI complex could be solved (79). Thus, peptide/class I interactions at the F pocket region seem to be sufficient to keep class I in a folded state. In agreement, short dipeptides mimicking the peptide C-terminus of high-affinity ligands support the folding of HLA-A*0201, displaying high peptide-receptivity (14). To stabilize peptide-free class I in a folded form independently from the peptide, the Springer group created a novel variant by introducing a disulfide bond to restrain the high flexibility of the F pocket region. The disulfide mutant showed an increased peptide and β_2_m affinity and bypassed the cellular quality control (80).

### MHC Class II

Physiologically, the question if peptide-free MHC class II proteins play a role in adaptive immunity is posed by studies indicating that unloaded MHC class II proteins are abundantly present on the surface of immature DCs. There, they are able to bind ligands from the extracellular milieu and activate T cells (81, 82). Two isomers of peptide-unloaded MHC proteins seem to exist, each displaying different kinetic properties (83). While the peptide-receptive empty isomer of DR1 binds peptide rapidly, the conversion to the non-receptive isomer within less than 5 min dramatically reduces the peptide-binding capacity of human DR (41, 84, 85). Studies using circular dichroism and size exclusion chromatography predicted a conformational change of peptide-free MHC class II upon peptide binding, and an increase in the overall stability (44, 68, 86). Peptide-free MHC class II proteins thus show a lower degree of helicity and an increased hydrodynamic radius compared to peptide-loaded MHC class II.

Carven and Stern studied ligand-induced conformational changes by selective chemical side-chain modification of peptide-free DR1 followed by mass spectroscopy analysis (87). The results of this study were inconsistent with a partly unfolded state of DR1 in the absence of ligand, but rather indicated a more localized conformational change induced upon peptide binding. However, empty MHC class II proteins harbor the hallmarks of partially unstructured peptide-binding domains when studied spectroscopically. NMR-spectra of the α-chain of such peptide-free MHC class II proteins barely display any signals for residues corresponding to the folded peptide-binding groove, indicating that the binding groove undergoes conformational exchange (46). Taken together, it appears likely that the empty binding groove dynamically samples different native-like conformations. Interestingly, similar to MHC class I, certain small molecules and dipeptides increase peptide-receptivity of peptide-free MHC class II proteins, presumably by preventing a “closure” of the binding groove (88, 89). In similar to the F pocket of MHCI, the predicted site of interaction is the most dominant pocket of the binding groove (P1).

In a MD simulation study combined with peptide-binding assays, it was shown that a well-conserved residue, βN82, which also contributes disproportionally to pMHCII stability (12, 90), is participating in the control of peptide receptivity (91). The authors suggested that the “non-receptive” state of peptide-free DR1 is induced by a molecular lock through the formation of a hydrogen-bond between DRβ N82A and DRα Q9. The observed narrowing of parts of the binding groove-flanking α-helices likely represents the trigger for such a clamped conformation. Using MD simulations, another investigation suggested a movement of the α51–59 region into the P1–P4 site of the binding groove in the empty state. In this state, the α51–59 region adopted a ligand-like conformation. In addition, an increased flexibility of the β2 domain as well as the β50–70 helical region were observed (92). A higher flexibility of the β58–69 helical region was also seen in MD simulations of the HLA-DR3 protein upon *in silico* peptide removal (93) (Table 1). Interestingly, this helical segment is also recognized by monoclonal antibodies designed to bind to the peptide-free conformation (81, 94).

**Table 1:** **Computational studies on MHC class I and II dynamics**.

MHC	Molecular dynamics parameters	Outcome	Reference
**MHC class I**			
HLA-B*2705, HLA-B*2709	GROMACS, OPLS-AA/L, TIP4P, 310K	Polymorphism of major histocompatibility class I (MHC class I) influences the dynamics of the binding groove and the bound peptide	Pohlmann et al. (95), Fabian et al. (96), and Narzi et al. (71)

HLA-A*02:01, HLA-B*44:02, HLA-B*44:05	Amber, parm03, TIP3P, 300K	Peptide-free MHC class I shows a varying flexibility at the F pocket region	Zacharias and Springer (9) and Sieker et al. (70)

HLA-A2:01, H-2K^b^	Amber, parm03, TIP3P, 300K	Prominent role of peptide C-terminus in long-range stabilization of MHC class I binding groove	Saini et al. (14) and Abualrous et al. (15)

HLA-B*44:02, HLA-B*44:05, H-2K^b^, HLA-B*2705, HLA-B*2709	(Amber, parm03, TIP3P, 300K) (GROMACS, Amber99SB-ILDN, TIP3P, 300K)	Dynamics of the F pocket region is important for MHC stability and modulates MHC class I tapasin dependance	Garstka et al. (21), Hein et al. (80), Abualrous et al. (22), Fleischmann et al. (37), and Fisette et al. (29)

BF2*15:01, BF2*19:01, HLA-B*44:02, HLA-B*44:05	GROMACS, Amber99SB-ILDN, TIP3, 300K	MHC intrinsic plasticity determines the bound peptide and can be modulated allosterically by tapasin	Bailey et al. (72, 73)

HLA-A2:01, HLA-B*3501, HLA-B*3508	(GROMACS, GROMOS43a1, SPC, 300 K) (NAMD, CHARMM22, TIP3P, 300K)	Peptide-MHC dynamics determine the T cell receptor (TCR) binding mode	Cuendet et al. (97) and Reboul et al. (98)

HLA-B*2705, HLA-B*2709, H-2D^b^	(GROMACS, OPLS-AA/L, TIP4, 310 K) (Amber, parm03, TIP3P, 350K)	Peptide-MHC interactions at the A pocket region modulate TCR recognition	Nurzia et al. (99) and Uchtenhagen et al. (100)

H-2L^d^	NAMD, CHARMM27, TIP3P, 310K	Peptide-receptive MHC class I shows a varying flexibility at the A pocket region	Mage et al. (74)

HLA-A2:01	(Normal mode analysis) (Modified Amber force fields, 300K)	Anti-correlative motion of residues in the binding groove is important for peptide binding “dynamic fit”	Nojima et al. (101)

**MHC class II**			
HLA-DR1, HLA-DR1	GROMACS, GROMOS9643a1, SPC, 298 K, GROMACS, GROMOS (ffG43a1), SPC, 310K	Peptide-free MHC class II show a large conformational flexibility around the P1 pocket	Painter et al. (92), Painter et al. (35), and Rupp et al. (91)

HLA-DR1 (1DLH)	GROMACS, GROMOS, 310K	Occupation of P1 with an amino acid side chains prevents the “closure” of the empty peptide binding site into the non-receptive state	Gupta et al. (89)

HLA-DR3, HLA-DR1	(Amber, parm03, TIP3, 300K) (GROMACS, GROMOS, SPC3, 310K)	Prominent role of peptide N–terminus in long-range stabilization of MHC class II binding groove	Knapp et al. (102) and Yaneva et al. (93)

I-Au, HLA-DR1 HLA-DR4	GROMACS, GROMOS, SPC3, 310K	Peptide-MHC dynamics influences T cells costimulation	Knapp et al. (103) and Knapp et al. (104)

HLA-DR4	GROMACS, GROMOS96, SPC, 310K	Different dynamics of soluble and membrane anchored pMHC	Bello and Correa-Basurto (105)
		The membrane anchored is more conformationally and energetically stable	

HLA-DR1	NAMD, CHARMM22, explicit water model, 298K	Conformational entropy of peptide binding to DR1 correlates with the DM-susceptibility	Ferrante et al. (106)

HLA-DR1	ACEMD, ff99SB, TIP3P, 310K	β-chain around peptide N-terminus and αW43 sample DM-bound-like conformations	Wieczorek et al. (46)

HLA-DR1	(Normal mode analysis) (Modified Amber force fields, 300K)	Anti-correlative motions in the binding groove is important for binding of long peptides “dynamic fit”	Nojima et al. (107)

HLA-DR1	(Normal mode analysis) (Modified Amber force fields, 300K)	The membrane-proximal domains of ~MHC class II modulate the dynamics of P1 pocket and have a greater influence on the binding groove than those of MHC class I	Nojima et al. (108)

*MHC I, major histocompatibility class I*.

Finally, it has to be noted that the timescale of the experimental descriptions of empty MHC molecules differs vastly from the theoretical studies. While the latter describe the initial events of conformational changes accompanying peptide removal, the experimental investigations observe the properties of the empty MHC species at or near equilibrium.

## Dynamic Features of Peptide-Bound MHC Complexes

While the study of empty MHC proteins is of theoretical and conceptual interest, nature has engineered the antigen-presenting system in a way that prevents the accumulation of isolated, non-peptide-bound MHC molecules. Endogenous peptides derived from the proteasome in case of class I or from the invariant chain in the case of class II first bind and eventually are replaced by antigenic peptide. This inherently dynamic process is enabled by intrinsic features of the MHC molecules and several studies suggest that pMHC complexes sample different and transient conformations dependent on the bound peptide and the allelic variant under investigation (46, 64, 71, 72, 95, 100, 106, 113, 114).

### MHC Class I

Changes in conformational dynamics in MHC class I are heterogeneously distributed along its peptide-binding groove, as suggested by both computational and experimental studies. For example, MD simulations showed a subtype-dependent conformational flexibility of the F pocket region. Residues 114 and 116 of the HC, at the bottom of the F pocket, and residues 74 and 77 from the α_1_-helix, engaging the peptide’s C-terminus, show an altered mobility in different MHC class I allotypes (9, 22, 70, 71, 96, 115, 116). Consistently, it was shown that the dynamics of the MHC class I binding groove was most profoundly affected by C-terminal residues of the peptide (15). In longer MD simulations, in addition to varying protein plasticity in the F pocket region, an enhanced sampling of conformations in the α_3_-domain upon peptide binding was observed (72, 73) (Table 1).

Experimental observations at the atomistic level, derived from NMR-based relaxation-dispersion experiments, have elucidated the peptide dependency of minor states on the stability of pMHCI complexes. Conformational fluctuations of different HLA-B*35:01 complexes were localized to the peptide-binding groove, including residues of the B, E, and F-pocket, but not in the IgG-like domains (113). Interestingly, the presence of minor conformations in pMHCI complexes (ranging from approximately >1 to 4.5%) could be positively correlated to the thermostability and surface presentation of the pMHCI complex under investigation, implying that a minor conformation considerably contributes to pMHCI stability. Similar, investigations of HLA-A*02:01 loaded with different peptides by HD exchange/MS and fluorescence anisotropy revealed that fluctuations within the binding groove depend on the ligand bound to MHC class I (112). Despite these ligand-sensitive changes in dynamics, the α_2_-helix showed a general higher flexibility than the α_1_-helix. The authors concluded that the observed variations in dynamics throughout the peptide-binding site could influence receptor engagement, entropic penalties during receptor binding, and the population of binding-competent states (see also Table 2).

**Table 2:** **Experimental studies on major histocompatibility complex (MHC) class I and II dynamics**.

MHC	Method	Outcome	Reference
**MHC class I**			
HLA-B*2705, HLA-B*2709	IR spectroscopy, crystallography	The heavy α-chain (HC) of B27:05 shows a higher flexibility than that of B27:09	Fabian et al. (109)
HLA-B*2705, HLA-B*2709	1H-15N-HSQC (NMR)	HLA-B27 polymorphism influences the β_2_m plasticity at the HC/β_2_m interface	Beerbaum et al. (110)
HLA-Cw*07:02	1H-15N-HSQC (NMR)	Peptide binding domains are “unstructured” in the peptide-free form	Kurimoto et al. (77)
HLA-B*2709	T1/T2 and HetNOE measurements	Regions of β_2_m remain flexible upon HC binding	Hee et al. (111)
HLA-A2	HDX/MS combined with fluorescence anisotropy	Fluctuations within the binding groove depend on the ligand bound to MHC class I	Hawse et al. (112)
HLA-B*35:01	NMR (relaxation-dispersion)	Stability of pMHC class I is determined by peptide-dependent fluctuations defining minor states	Yanaka et al. (113)
**MHC class II**			
HLA-DR1	HDX combined with mass spectroscopy	3_10_ helix shows a conformational lability	Painter et al. (35)
		DM-susceptible conformations show weakened interactions around the P1-pocket	
HLA-DR1	NMR combined with crystallography	Peptides can bind in an bidirectional mode to DR1	Gunther et al. (57)
HLA-DR1	SACS, NMR, crystallograpy	Susceptibility to HLA-DM depends on a dynamic conformation of pMHC class II	Yin et al. (64)
HLA-DR1	NMR detected HDX, HSQC spectra	Dynamics in helical segments and and αS2/S4 strand of binding groove	Wieczorek et al. (46)
		Peptide binding domains are “unstructured” in the peptide-free form	

*HDX, hydrogen/deuterium (H/D) exchange; HSQC, heteronuclear Single Quantum Coherence; SAXS, small-angle X-ray scattering*.

Another study also showed that β_2_m seems to sense allelic as well as peptide-induced conformational variations and accommodates to them, showing a high degree of plasticity within the inter-domain interface with the HC domains (110). NMR-chemical-shift changes of complexes were most pronounced in the region close to the F-pocket. By comparing the dynamics in the ns–ms timescale of HC-bound and free β_2_m, Hee et al. demonstrated that most residues gain rigidity upon HC binding (111). Nevertheless, three sites (region around His31, site around Asp53 and Lys58, and region around Ser88) remained flexible in the mature complex. Interestingly, His31 and Ser88 are located underneath the F-pocket, which stability is known to be important for tapasin function. Moreover, Lys58 and Ser88 are also known to interact with several other proteins, including the natural killer cell receptors Ly49A CD8 and LIR1 (117–119). Conformational sampling of these regions could thus be critical for the interaction with these receptors.

### MHC Class II

A similarly heterogeneous picture in regards to altered binding groove dynamics can be observed for MHCII: by dissecting the thermodynamics of peptide MHC class II interactions, Ferrante et al. conclusively demonstrated how dynamics of pMHCII complexes are linked to peptide affinity and DM-susceptibility (106). Using different biophysical techniques and MD simulations, the authors showed that peptide binding events can be driven by a considerable proportion of conformational entropy (if enthalpic interactions are less favored). MD simulations suggest that peptide-dependent conformational fluctuations involve alterations of α-chain residues DR1α-43–54 and β-chain residues 63–68 and 79–90. Wieczorek et al. performed H/D-exchange measurements in combination with NMR spectroscopy to obtain residue-specific experimental information about the stability of individual secondary structure elements. Several regions undergoing conformational fluctuation even in highly stable pMHCII complexes were thus revealed. These fluctuations are confirmed by extensive (~100 μs) MD simulations and Markov model analyses that reveal transient conformations with obvious relevance for the peptide-exchange pathway (see below for details) (46). In particular, the highest lability was seen in DRα 46–62, in parts of β-strands s2–s4 sitting underneath the N-terminal part of the α_1_ helix, β65–93 and several loops connecting the β-strands of the peptide-binding site. Interestingly, this experimental piece of evidence is also in line with the global B-factor analysis presented here (Figure 2B); a certain degree of dynamics thus seems to already be encoded even in the context of a high affinity pMHC complex. Earlier on, it was shown by HD-exchange/MS measurements that conserved peptide–MHC class II contacts (H–bonds) are strong at the P1 pocket-proximal site of the peptide (especially position βN82) in highly stable immunodominant complexes, which is in agreement with previous biochemical studies (12, 35, 90). However, local destabilization induced by a point mutation in the DR1 complex and the use of a different ligand (DR1-αF54C/CLIP) strongly enhanced fluctuations of the peptide and especially weakened contacts around the P1 site (35). This implies that weakening interactions by substitutions in the peptide or MHC (allelic variation) would have an influence on conformational fluctuations that correlate with DM-susceptibility.

## MHC Dynamics During Peptide Exchange

While the studies described in the previous section unambiguously demonstrate the dynamic features of pMHC complexes, the question arises naturally in how far these properties translate into peptide exchange. For pMHCII complexes experimental progress has been made in identifying intermediate or transient conformations of pMHCII with regard to catalyzed or intrinsic peptide exchange (13, 31, 32, 35, 46). For pMHCI molecules the atomistic description of structural changes during peptide exchange mostly relies on MD simulation studies, supported by mutational analysis and circumstantial biophysical evidence (14, 22, 37, 73).

### MHC Class I

Within cells, tapasin is a key protein that mediates the binding of high-affinity peptides to most class I proteins. To date, there is no crystal structure of the tapasin/MHC class I complex. Based on mutational studies, however, two regions have been shown to be essential for tapasin interaction with the HC of MHC class I: a loop in the α_3_ domain (residues 222–229) and a part of the α_2_ domain (residues 128–137) (17, 23, 25, 120, 121). Two major functions have been proposed for tapasin: (i) a chaperone-like stabilization of empty class I proteins (20–22, 24, 122) and (ii) a peptide-editing function through peptide-exchange catalysis (26, 123, 124). Several computational models have been published to describe the mechanism of action of tapasin on MHC class I (28, 29, 121). Most researchers agree on the importance of the F-pocket region for peptide exchange (14, 21, 37, 80, 125). However, the association of F-pocket dynamics and the peptide-exchange mechanism remain a matter of debate. So far, dynamics in the F-pocket region in the presence of peptide have not revealed any significant conformational exchange phenomena in most MD simulations (Table 1). Recently, longer MD simulations (microsecond timescale) of tapasin/pMHCI complexes indicated that binding of tapasin to HLA-B*44:02 accelerates the dissociation of low-affinity peptides (29, 37). Using a computational systems model (73, 126), it was shown that peptide exchange seems to depend on the opening and closing rate of the binding groove in the presence of peptide. According to this study, the pMHCI opening rate is peptide-dependent, but pMHCI closing is allele-dependent. Consequently, a low-affinity peptide complex would display fast opening rates, but only if the MHC allele variant has an F-pocket signature (more plasticity) that allows for fast closing in the presence of a high-affinity peptide (as B44:05), it would lead to efficient peptide exchange in the absence of catalyst. Allele variants with a rigid F-pocket conformation (as B44:02) in contrast depend on tapasin to sample the necessary conformational states to close the binding groove quickly.

It has to be considered that as a default, tapasin is present in the cell and that it may also provide the necessary function as a chaperone to prevent the collapse of empty MHC class I molecules into a non-receptive state (29, 127) as it is experimentally measured in tapasin-deficient cells (21, 22). However, in the absence of a crystal structure of the tapasin/MHC class I complex, it is difficult to rationalize the dynamics with regard to tapasin binding and exchange.

### MHC Class II

Two seminal studies made it unambiguously clear that HLA-DM recognizes complexes showing a P1-destabilized conformation (13, 31). However, since DM-susceptible structures rarely show any of the changes present in the DM-bound structure (e.g., folding of α-46–55 into an α-helix and unfolding of C-terminal β-helix), the question of the conformational prerequisites for DM binding arises. As mentioned previously, an important study indeed demonstrated that HLA-DM attacks pMHC class II complexes at a site of conformational lability, the 3_10_-helical region (35). This segment, together with a neighboring unstructured segment (α52–55) folds into an α-helix when bound to DM (31). Interestingly, increased fluctuations of this region could be observed by other computational and experimental studies, implying the existence of higher conformational entropy within this region (46, 106). Noteworthy, the α-helix in the region β86–β91 opens up to a certain degree in the DM-bound structure of DR1. However, a considerable influence of P1-remote sites on conformational dynamics and DM-dependence was recently demonstrated (64). In this study, alteration of P9-pocket/peptide interactions influenced dynamics of the pMHCII, likely in regions relevant for DM binding. The authors concluded that the key determinants for HLA-DM recognition are conformational dynamics present in HLA-DR1. Similarly, and as already mentioned above, Ferrante et al. explained the relationship between entropic penalties and DM binding in a thermodynamic context (106). According to their experimental and computational results, higher conformational entropy of pMHCII complexes correlates with DM susceptibility.

A recent study by our group in the MHC class II field explored internal motions of pMHC class II molecules along the conformational peptide-exchange pathway in a more conceptual model (46). Using NMR/HDX (hydrogen deuterium exchange) and MD simulations of over 200 μs in total, followed by Markov State model (MSM) analysis (128–132), we have identified transient conformations relevant for the DM-catalyzed and non-catalyzed (spontaneous) peptide-editing process (46). In agreement with the general view, the catalyzed pathway depends on the particular destabilization of the region surrounding the P1 pocket, sharing in part features of MHC class II bound to DM. More specifically, it has been suggested for MHC class II that pMHC complexes have to sample P1-pocket-destabilized conformations to allow for HLA-DM binding (13).

The non-catalyzed pathway, however, was correlated to the ground state of the pMHCII complex and, therefore, is directly correlated with thermodynamic stability. Indeed, it was shown that, removal of two hydrogen bonds between β82N of the MHC class II and the backbone of the peptide in the mutant DR1βN82A drastically reduces stability and, at the same time, dramatically enhances non-catalyzed peptide exchange. Nevertheless, binding to HLA-DM is also enhanced for the βN82A mutant, leading to the somewhat paradoxical finding that an MHCII molecule might bind tightly to a catalyst that it does not need for exchanging peptide. This can be best conceptualized when assuming that the βN82A mutant of HLA-DR1 in addition to increased spontaneous exchange more frequently samples a rare conformation along the pathway of catalyzed exchange. MD simulations in conjunction with MSM analysis indeed show that an excited state structurally correlated with features of the HLA-DM bound conformation. This excited state was seen to be significantly more frequently sampled in the mutant compared to WT HLA-DR1.

However, a similar intermediate state can be defined for the very stable WT protein, where peptide release from the pockets was not mandatory for the observation of the early intermediates. Thus, if the pMHCII forms a stable complex, the peptide editing depends on the population of rare conformations that can be selected by the catalyst DM for binding. This study demonstrated the critical importance of residues 80–93 of the β1 helix for catalyzed exchange, suggesting that β1 helical unfolding is critical for the rearrangement of this segment as it is observed in the DR1-DM structure (31). As the study shows, mutations in the β1 helix (e.g., βE87P) designed to specifically destabilize the C-terminal part of the β1 helix without disrupting H-bonds to the peptide, are able to over-proportionally shift the dynamics toward the HLA-DM-dependent pathway (46).

In conclusion, this model helps to reconcile discrepancies in the hypothesized correlations of peptide affinity, pMHC stability, DM susceptibility, and catalytic effect (133).

## Conclusion and Outstanding Questions

Major histocompatibility complex proteins are encoded by oligogenic and highly polymorphic genes and most polymorphisms map to the regions important for peptide binding. The pMHC complexes display various degrees of flexibility along the binding groove, and these dynamic features seem to correlate with the propensity for peptide exchange. Of interest is the fact that tapasin and DM both bind their MHC targets in regions of enhanced dynamics. Short, destabilized helical segments together with their adjacent structural elements seem to represent the requirements for transient binding of the respective catalyst. The degree of these local flexibilities can be correlated to a higher dependence of a particular pMHC complex on tapasin or DM. Polymorphic substitutions might not only change the binding preference for certain ligands but also the overall stability and dynamics of the corresponding allelic variants. In turn, this will affect the conformational ensemble recognized by the peptide editors and in principle should be able to explain why certain alleles seem to possess a generally lower taspasin or DM dependence. In this way, MHC molecules may become a paradigmatic example of how differences in the dynamic landscape of protein complexes translate into distinct functional outcomes of physiological relevance.

**Figure 4:**
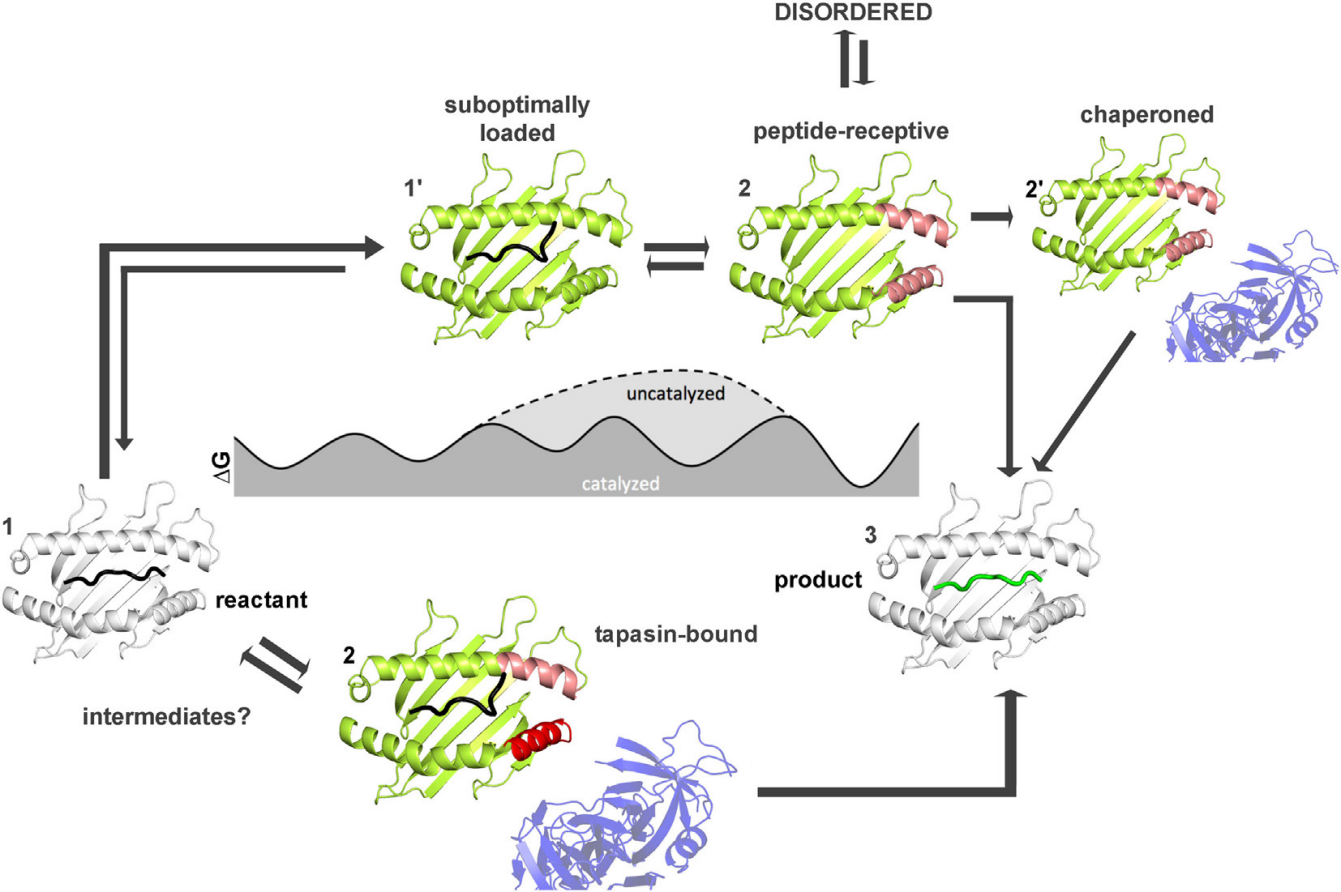
**Thermodynamic model for peptide exchange of major histocompatibility complex (MHC) class I**. Peptide–MHC class I (pMHCI) complexes can follow two mechanistic pathways for peptide exchange starting from pMHCI ground state (state 1). In the tapasin-catalyzed pathway, tapasin modulates conformational changes in the α2-1 helix (red) of the F pocket region (pink) and the α3 domain (not shown) that accelerate the kinetics of peptide dissociation (state 2) and the loading of a high-affinity peptide (3). More intermediates states (between state 1 and state 3) need to be identified by computational studies and/or NMR and X-ray crystallography. In the non-catalyzed pathway, the peptide dissociates from the sub-optimally-loaded intermediate state (state 1′). The resulting empty MHC molecule shows subtype-dependent dynamics (especially at the F pocket region, pink) and thus can exist in a stable peptide-receptive form (state 2′) or in an unstable form (state 2″) that is chaperoned by tapasin for peptide binding. The structures used in states 1 and state 3 were modified from PDB: 1UXS (shown in white). The models used in states 1′, 2, 2′, and 2″ represent suggested states by computational and experimental studies (shown in limon).

**Figure 5:**
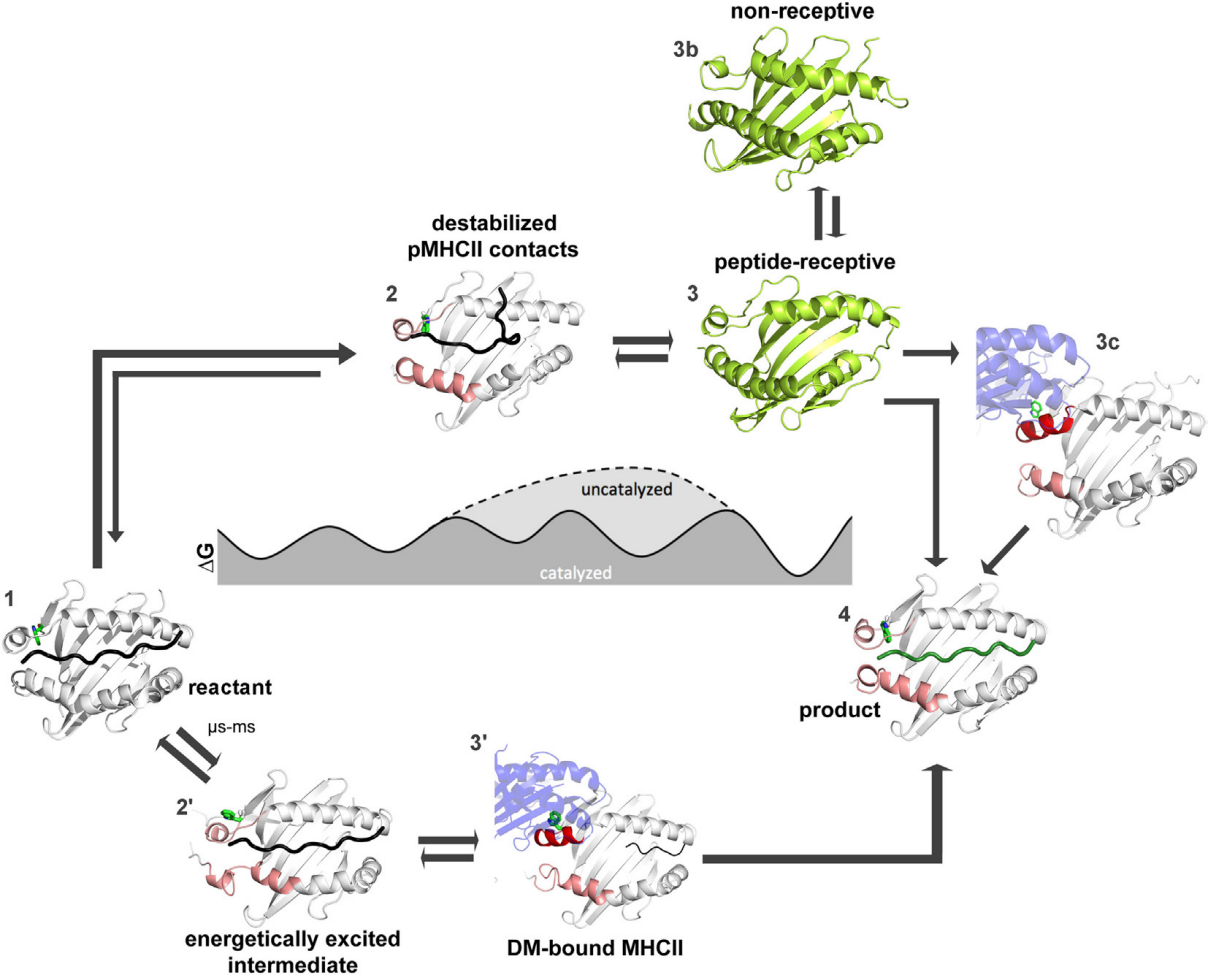
**Thermodynamic model for peptide editing of major histocompatibility complex class II**. pMHCII complexes can follow two mechanistic pathways for peptide exchange. The DM-catalyzed route requires multi-step transitions starting from the pMHCII ground state (1). This includes initially an out-flip movement of αW43 or the destabilization of β80–93 region *via* spontaneous conformational sampling of rare conformations (state 2′). DM would preferably select for conformations that are sampled on longer timescales and which show both, an out-flip movement of αW43 and a destabilized β80–93 region. Binding of DM to the energetically excited intermediate (which shows in part features of the DM-bound state) would then induce further rearrangements in the 3_10_-helical region (state 3′) and thereby accelerate peptide-release. Binding of peptides which can displace the stabilizing interactions complete the peptide exchange process (state 4). Spontaneous (non-catalyzed) peptide exchange depends on the intrinsic stability of the pMHCII complex and does not rely on the sampling of rare conformations (state 2). Binding of a new peptide would likely require dissociation of the bound peptide, leading to the empty state (state 3) which rapidly converts into the non-receptive state (state 3b) but can also be chaperone by DM (state 3c) in order to allow for high-affinity peptide binding (state 4). Structures used in state 1, 3′, and 4 were derived from PDB: 4QXA, 4FQX, and 1DLH, respectively. Cartoons shown in 2, 2′, 3, and 3b were derived from molecular dynamic simulations (46, 91).

How far have we come and what has to be done to achieve this goal? Figures 4 and 5 summarize the findings described in this review and also emphasize the most daunting questions in the field that need to be answered in order to formulate a unifying concept of antigen exchange. What seems to be clear is that both type of MHC molecules can exchange peptide along two distinct pathways, with the ratio of spontaneous versus catalyzed exchange certainly being different for the allelic protein variants and pMHC complex. While dynamics often correlates with thermodynamic stability, it has yet to be seen which type of motions are critical for catalysis and which structural elements are indispensable for these transitions to occur. For MHC class II molecules, the structure of the MHCII–DM complexes provides a cornerstone (31), and the early intermediates (μs-ms timescale) toward the DM-bound form could be defined (35, 46) (Figure 5). However, there are no structural insights about the replacement of DM by incoming peptide, thus requiring experimental and simulation strategies to follow the fate of DM-prebound MHCII molecules. In the case of MHCI, a tapasin-class I complex structure is required in order to provide a reliable framework for further experimental and theoretical studies. Similarly, characterization of empty MHC molecules will certainly aid in defining the dynamic modes that are explored by the peptide-binding domains. Since it has been shown that empty MHC molecules can be rescued by the chaperoning function of the exchange catalysts (134, 135) and thus the dynamics that occur upon peptide exchange are likely to show features of the empty state. It seems, therefore, highly desirable to compare the two systems on time scales down to a few milliseconds.

For both MHC classes, more sophisticated NMR experiments capitalizing on selective amino acid side-chain labeling protocols are probably required and methods relying on CEST or relaxation dispersion should be able to yield more direct information on the anticipated intermediate states (136, 137). So far, in-depth NMR experiments are restricted to certain stable pMHC complexes and the investigation of other alleles have been hampered by the in-availability of other variants such as the disease-relevant DQ alleles. There is a need to expand the experimental basis of dynamically investigated pMHC complexes in order to test the predictions made on the basis of the dynamic features of just a few alleles. Solutions are most likely to come from protein engineering approaches in combination with the use of different expression systems. The increasing importance of MD simulations arises from the fact that micro-to-milli-second simulations in combination with Markov State Modeling will become more of a standard in the field. This is essential, because the critical intermediates of antigen exchange seem to be populated at this time scale. Once we are able to conceptualize conformational peptide exchange, we will be in the position to better predict MHC peptide occupancies in the context of cellular editing mechanisms and we will understand and be able to manipulate the action of small molecules or biological macromolecules that modulate peptide exchange.

## Author Contributions

MW, EA, and CF designed and wrote the manuscript. JS, MÁ-B, SS, and FN critically read, discussed, and edited the content prior to submission.

## Conflict of Interest Statement

The authors declare that the research was conducted in the absence of any commercial or financial relationships that could be construed as a potential conflict of interest.
